# Didehydrophenylalanine, an abundant modification in the beta subunit of plant polygalacturonases

**DOI:** 10.1371/journal.pone.0171990

**Published:** 2017-02-16

**Authors:** Kjell Sergeant, Bruno Printz, Annelie Gutsch, Marc Behr, Jenny Renaut, Jean-Francois Hausman

**Affiliations:** 1 Luxembourg Institute of Science and Technology (LIST), Environmental Research and Innovation (ERIN) department, Esch-sur-Alzette, Luxembourg; 2 Université catholique de Louvain, Earth and Life Institute Agronomy, Groupe de Recherche en Physiologie Végétale Louvain-la-Neuve, Belgium; 3 University of Hasselt, Centre for Environmental Sciences, Environmental Biology, Diepenbeek, Belgium; Fachhochschule Lubeck, GERMANY

## Abstract

The structure and the activity of proteins are often regulated by transient or stable post- translational modifications (PTM). Different from well-known, abundant modifications such as phosphorylation and glycosylation some modifications are limited to one or a few proteins across a broad range of related species. Although few examples of the latter type are known, the evolutionary conservation of these modifications and the enzymes responsible for their synthesis suggest an important physiological role. Here, the first observation of a new, fold-directing PTM is described. During the analysis of alfalfa cell wall proteins a -2Da mass shift was observed on phenylalanine residues in the repeated tetrapeptide FxxY of the beta-subunit of polygalacturonase. This modular protein is known to be involved in developmental and stress-responsive processes. The presence of this modification was confirmed using in-house and external datasets acquired by different commonly used techniques in proteome studies. Based on these analyses it was found that all identified phenylalanine residues in the sequence FxxY of this protein were modified to α,β-didehydro-Phe (ΔPhe). Besides showing the reproducible identification of ΔPhe in different species arguments that substantiate the fold-determining role of ΔPhe are given.

## Introduction

Post-translational modifications (PTM) are an essential part of the repertoire by which living organisms modulate the properties of proteins. Besides frequently occurring modifications such as glycosylation, phosphorylation or proteolytic processing, the presence of a PTM identified in one or a limited number of different proteins originating from a wide range of species is not unheard of. The best-known example is the diphtamide modification exclusively found on a specific histidine of the eukaryotic elongation factor 2 in all studied eukaryotes [1]. These rare, protein-specific PTMs and their function often remain elusive, however based on biological logic a significant physiological importance must be attributed to them. Without such physiological importance, the evolutionary conservation of a rare, highly-specific modification and the enzyme that catalyses the modification (or set of enzymes as for dipthamide [2]), would be in contradiction with the principles of biological energy conservation and evolution itself.

The influence that post-translational modifications have on protein function generally is the consequence of a primary influence on the three-dimensional structure of the protein. This structure-determining effect is obvious for some side chain modifications, such as those able to covalently link distant parts of the polypeptide chain or different polypeptides (cystine or dityrosine bridges [3]). The influence of other modifications on protein fold may be more subtle [4,5], but therefore not of less importance [6,7]. Since the changes in weak interactions induced by a small chemical modification can only at specific points in a fold result in the required structural shifts, the modified amino acids are generally conserved. In yeast it was found that the variability of phospho-sites is more constraint than that of surrounding sequences [8]. Similar observations were done when comparing phospho-sites from *Arabidopsis* and rice [9].

The beta subunit of polygalacturonase (βPG) is extensively studied for its implication in fruit ripening [10,11]. The proposed role of the protein is to physically limit the access of pectin hydrolases to pectin by strongly binding the cell wall polysaccharide [12], although a decreased pectin content is observed in βPG-overexpressing rice [13]. The protein is synthesized as a 3-domain precursor: a N-terminal domain containing a signal- and pro-peptide and a C-terminal BURP domain of unknown function but essential for phenotype effects [14,15]. The central domain is mainly composed of 14-amino acid long repeats starting with phenylalanine [11]. The active protein, as it is isolated from the cell wall, is composed exclusively of this middle domain and it is known that most of the Phe-residues of this protein are modified [11]. However the nature of the modification remained unknown. Using gene expression analysis the gene is found to be highly expressed in different tissues and changes in the expression of βPG-encoding genes are observed when plants are exposed to constraining conditions [13,16]. Nonetheless, changes in the abundance of this protein where never reported using proteome analyses.

Here we report the first description of didehydrophenylalanine (ΔPhe) in βPG isolated from the alfalfa cell wall, and in intact proteins in general. The occurrence of this modification in the βPG of other species was done by the reanalysis of datasets from published studies [17]. This approach allowed confirmation based on experiments that used all frequently-used techniques in proteomics. Although not high in number, βPG was only identified with the phenylalanine residues in the sequence FxxY modified to didehydrophenylalanine and no other protein was identified that has the same modification. These observations allow postulating that a, previously unknown, enzymatic function in plant cells converts phenylalanines to dehydrophenylalanine. It is furthermore hypothesized that the main function of these modifications is to direct the structure of the protein, thereby allowing the tight binding of βPG to pectin and the catalytic subunit of polygalacturonase.

## Results

**Fig 1:**
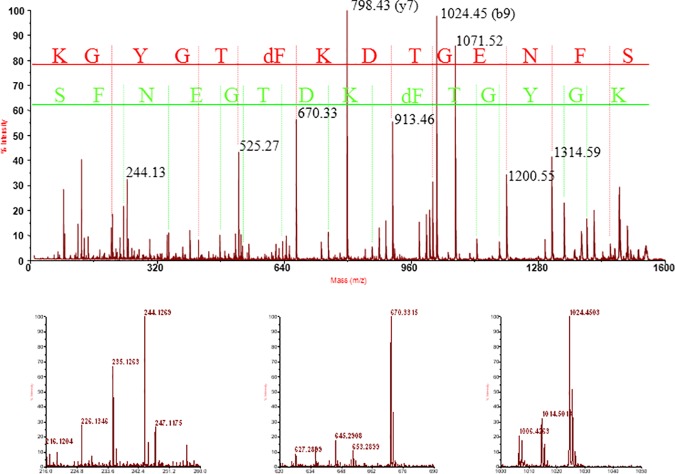
MS/MS spectrum of the precursor at m/z 1548.6924. The peptide was identified as SFNEGTDKFTGYGK from the Cannabis sativa polygalacturonase non-catalytic protein (NCBI EST database GI:156080210). The upper panel shows the MS/MS spectrum with y- and b-ions indicated respectively in red and green. The one-letter code is used for the amino acids and dF indicates a didehydrophenylalanine. The lower spectra illustrate the specificity of the modification. While no mass shift is observed for the Phe closest to the N-terminus, illustrated by the lack of secondary peak at 233 for the b2-ion shown in the left panel, the more C-terminal Phe is completely modified as illustrated for the y6- and b9-fragment in the central and right lower panel.

**Fig 2:**
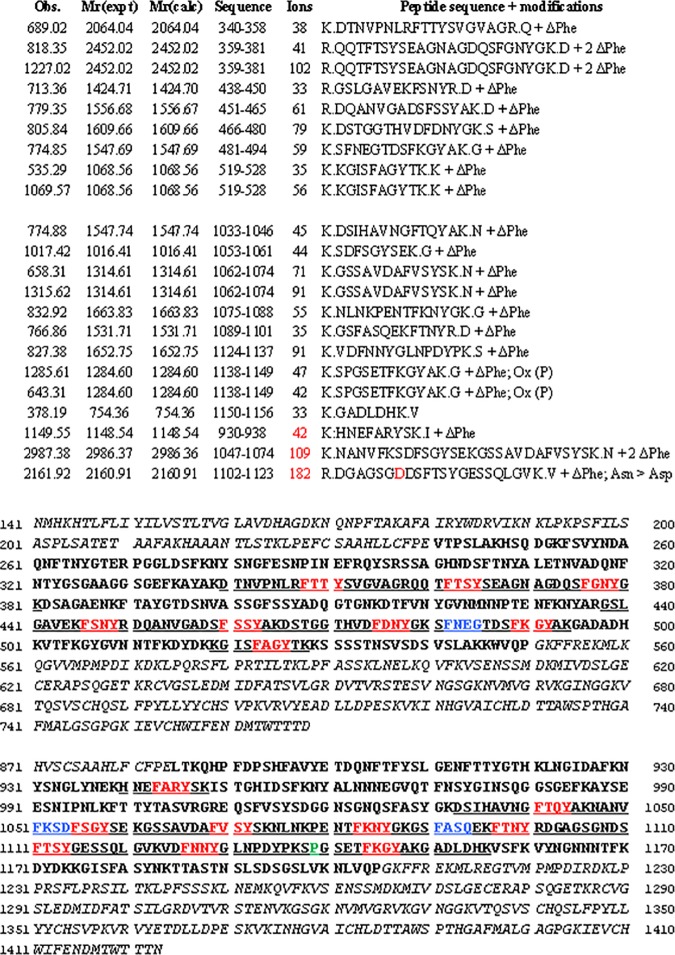
Identified peptides and sequence coverage for the contig 53836 (AGED database). The upper panel shows the identified peptides, mainly based on a database search of the dataset ft2011092714 (http://dx.doi.org/10.6084/m9.figshare.100494)[19]. Peptides uniquely found in in-house datasets are indicated in red. The lower panel shows the translated contig, it contains two sequences coding for different βPG homologs. The signal- and propeptide (residue 1–110) as well as the two C-terminal BURP–domains are indicated in italics. The identified peptides are underlined and the repetitive tetrapeptide FxxY with modified phenylalanine in red. Those phenylalanine residues that were identified as not modified are indicated in blue. The proline that is reproducibly found to be oxidized is in green. Similar observations were done for other contigs.

During the optimization of a protocol for the isolation of cell wall proteins [18], a 2Da mass shift was observed on most but not all Phe-residues of the βPG of alfalfa (*Medicago sativa*). Reanalysis of an in-house generated 2D-gel based dataset from *Cannabis sativa* hypocotyls, extracted using a completely different protocol confirmed the observation of this mass shift on Phe-residues of the homologous hemp protein (Fig 1 and Table 1). Independent datasets from alfalfa cell wall protein extracts, gel-based with MALDI analysis or gel-free with ESI analysis (PRIDE archive PXD001927) [18], and of a publicly available dataset [19], were reanalysed against the AGED database. These analyses confirmed that most of the phenylalanine residues of the different homologous alfalfa proteins are quantitatively modified with the -2Da mass shift. No similar mass shift was observed for other proteins. In Fig 2 the identified alfalfa sequences from both datasets corresponding to contig 53836 (AGED annotation) are represented.

**Table 1:** Peptides from βPG identified in *Arabidopsis*, cannabis and maize.

***Cannabis sativa*** *hypocotyls*						
gi|156080210 (EST) polygalacturonase non-catalytic protein (*Cannabis sativa*)						
	**Obs.**	**Exp.**	**Cal.**	**ppm**	**Ions**	**peptide sequence**
	1548.69	1547.69	1547.69	-2.67	101	K.SFNEGTDKFTGYGK.G + ΔPhe
	1583.71	1582.70	1582.70	-0.28	77	K.SSNAEQINFNNYGK.S + ΔPhe
	3000.38	2999.38	2999.39	-5.18	29	R.QGGSDQFKNYSPGENIPVDSFRRYSR.D + 2 ΔPhe
***Arabidopsis thaliana***						
**Different tissues [20]**						
gi|1762584 polygalacturonase isoenzyme 1 beta subunit homolog [*Arabidopsis thaliana*]						
Dataset	**Obs.**	**Exp.**	**Cal.**	**Da**	**Ions**	**peptide sequence**
3322	1205.8	2409.59	2408.07	1.51	70	K.VNFVNYGQSFNPGSETFTGYGK.G + 2 ΔPhe *
3335	1205.01	2408.01	2408.07	-0.07	46	K.VNFVNYGQSFNPGSETFTGYGK.G + 2 ΔPhe *
3337	1205.69	2409.36	2408.07	1.29	77	K.VNFVNYGQSFNPGSETFTGYGK.G + 2 ΔPhe *
**Soluble proteome of cell suspension cultures [21]**						
gi|1762584 polygalacturonase isoenzyme 1 beta subunit homolog [*Arabidopsis thaliana*]						
	**Obs.**	**Exp.**	**Cal.**	**ppm**	**Ions**	**peptide sequence**
	679.80	1357.59	1357.58	4.46	49	K.ANVGDDSFSSYAK.D + ΔPhe
***Zea mays***						
**dataset Maize_endosperm-10-try-150ug-2D29-122109-LTQ3 (22;23)**						
gi|195613864 polygalacturonase-1 non-catalytic beta subunit precursor [Zea mays]						
Fraction	**Obs.**	**Exp.**	**Cal.**	**Da**	**Ions**	**peptide sequence**
20	553.59	1657.75	1657.65	0.098	40	R.DDGNVGDDRFTSYAK.G + ΔPhe
	829.75	1657.49	1657.65	-0.16	48	R.DDGNVGDDRFTSYAK.G + ΔPhe
23–25	1117.84	2233.67	2233.22	0.44	108	R.SFASYSQEANHGENGFSGYGK.N + 2 ΔPhe
	745.83	2234.47	2233.22	1.25	60	R.SFASYSQEANHGENGFSGYGK.N + 2 ΔPhe
27	326.11	650.21	649.31	0.89	30	K.SGVDFK.G + ΔPhe
28	693.23	2076.67	2076.19	0.48	46	FRSYGAGGNAGVDTFKNYR + 2 ΔPhe
**dataset Maize juvenileleaf-1-Try-3mg-MCX-1500ugCeO2-Elu-2d19-030210-LTQ3 (22;23)**						
gi|195613864 polygalacturonase-1 non-catalytic beta subunit precursor [Zea mays]						
Fraction	**Obs.**	**Exp.**	**Cal.**	**Da**	**Ions**	**peptide sequence**
14	554.13	1659.28	1657.65	1.63	28	R.DDGNVGDDRFTSYAK.G + ΔPhe
	830.12	1658.23	1657.65	0.58	43	R.DDGNVGDDRFTSYAK.G + ΔPhe
17/18	1117.86	2233.71	2233.22	0.48	80	R.SFASYSQEANHGENGFSGYGK.N + 2 ΔPhe

* For these peptides the accepted mass error on the original data surpasses the mass shift induced by the modification, making that partial modification was found. Manual inspection of the MS/MS spectra however indicates that both phenylalanines are modified (Figure A in S1 File), the result presented corresponds to the search with a corrected precursor mass.

Deglycosylation of the protein with PNGaseF was done in order to increase the sequence coverage, however no additional sequence was found. Although a high sequence coverage was obtained for what is considered as the active protein, no peptides corresponding to sequence outside of the repetitive part are identified. All the identified phenylalanines in the sequence FxxY were observed as modified with a mass shift of -2Da, while other phenylalanines were not. The observation of a residual mass of 145Da instead of the 147 for unmodified phenylalanine (Fig 1), indicates the presence of an α,β-didehydrophenylalanine (ΔPhe), likewise called dehydrophenylalanine, a modified amino acid not previously identified in proteins.

To confirm the modification in other species, datasets were downloaded from public repositories or kindly provided by other researchers (Table 2). In most of these datasets the protein was not identified nor was ΔPhe reliably observed in any other protein, even when defining ΔPhe as variable modification. The protein and the modification were however found in a dataset from *Arabidopsis* [20] (Pride archive PRD000044), and more specifically in the data-files from the SDS fraction of open flowers (3322), the soluble fraction from seeds (3335) and the urea fraction of flower carpels (3337). The protein was likewise identified in a dataset of soluble proteins from *Arabidopsis* suspension cultures [21] (Table 1). During the analysis of datasets from different maize organs/tissues [22,23] all the phenylalanines of βPG that were identified were also modified. All the peptides in which this modification was found are represented in Table A in S1 File. Contrary to the data from alfalfa, maize and, to a lesser extent, cannabis only one peptide with a score above the threshold of significance was found in these *Arabidopsis* samples. For the alfalfa analyses, both internal and by Verdonk *et al*., the protein was only found in the LiCl fraction, the fraction containing proteins tightly associated with the cell wall matrix. No such targeted extraction was used for the *Arabidopsis* or maize samples. However since using completely different extraction protocols results in the same modification, the possibility that it is an artefact due to a specific extraction protocol, in casus with LiCl, is excluded.

**Table 2:** External datasets that were reanalysed.

Species	Tissue/Organ	Extraction protocol	βPG	Ref.
Alfalfa	Stem	CaCl_2_ and LiCl	+	[19]
Arabidopsis	Different tissues	Optimized for each tissue	+	[20]
Arabidopsis	Secretome	TCA/aceton precipitation	+	[21]
Zea mays	Different organs/tissues	Precipitation with 0.2 mM Na_3_VO_4_ in methanol		[22,23]
Arabidopsis	Stem	CaCl2-extraction	-	[24]
*Glycine max*	Seed testa	Phenol two phase system	-	[25]
Tomato	Secretome	-	-	[26]
*B*. *distachyon*	Grains	CaCl_2_ and LiCl	**-**	[27]
Arabidopsis	Leaf	CaCl_2_ and LiCl + glycoprotein enrichment	-	[28]

A proline, indicated in green in Fig 2, was likewise reproducibly found to be hydroxylated in different experiments using gel-based and gel-free proteome methods. Proline hydroxylation is a well-known modification in collagen wherein it is essential for the stabilization of the triple helical structure [29]. The modification is known for other cell wall proteins, it was for instance recently found in class III peroxidases [30].

In a comparative study using 2D-DiGE on the impact of Cd-exposure on the cell wall proteome of alfalfa, βPG was again found in the LiCl fraction (A. Gutsch, manuscript in preparation). Quantitative analysis of the acquired gel images revealed that 87 spots changed significantly in intensity when comparing cadmium-exposed with control samples. Of these 87 spots, 18 contained different βPG isoforms. This confirms the implication of this protein in the response to environmental constraints as previously observed by gene expression analysis in different systems.

## Discussion

By using a set of methods covering the entire range commonly used in proteomics, our analyses reveal that in mature βPG all identified Phe residues in the sequence FxxY are modified. The 2Da loss from phenylalanine, and thus a residual mass of 145Da instead of 147Da (Fig 1), indicates the presence of α,β-didehydrophenylalanine (ΔPhe), likewise called dehydrophenylalanine. An example of the sequence coverage attained for a specific contig from alfalfa is given in Fig 2. Taking our entire dataset into account, it can be assumed that those Phe residues in the sequence FxxY that were not identified are likewise modified. This assumption is in agreement with the amino acid analysis published by Zheng et al. (Table 2 in the article, [11]). When expressed on the basis of 100 amino acids, Zheng et al. identified one unmodified phenylalanine. Calculated for the full length of the tomato homologue, 288 residues from residue 109 to 396 of NCBI entry 350538029, this makes 2.6 unmodified phenylalanine residues out of the 23 predicted based on nucleotide sequence. Two of the 23 phenylalanines in this 288 amino acid long polypeptide are not found in the sequence FxxY. Given the limits of amino acid analysis the calculated value of 2.6 is a close enough approximation to support the claim that all phenylalanines in the sequence FxxY are modified.

This high number of modifications also explains why βPG was to our knowledge never before identified in proteome studies although it is regularly identified as being highly expressed [31]. When the expression level of for instance the gene P92990, one of the βPG-homologues in *Arabidopsis*, in different tissues is visualized with Genevisible it is found to be high in all tissues. Furthermore, this gene is classified as ‘expressed at very high level’, 4.2 times the average gene, by Aceview. Our analysis of published datasets shows that the protein is indeed present in some of these samples and that it can be identified if the modification of phenylalanine is included in the search parameters. The fact that the protein is not identified in most of these datasets does not contradict the above postulated claim, but can be explained by the strong association of the protein with the cell wall matrix. Furthermore, the conservation of the FxxY-tetrapeptide in the 14 amino acid long repeat in homologous proteins from other plant species allows postulating that the modification will also to be found in proteins from other species. Therefore, the description of ΔPhe as modification in βPG will allow generating new biological knowledge on the regulation of this protein and its implication in biological processes in other experimental setups.

For instance our analysis, allowing this modification as variable, of the different in-house generated gel-based studies but also of the LC-MS/MS dataset from Verdonck et al [19], results in the identification of βPG among the highest scoring proteins (Fig 2). In ongoing studies we have furthermore found this protein to be differentially abundant during stem development and in response to exposure to Cd (unpublished data). These form the first proteome-level indications that βPG is involved in these processes. These observations are however in agreement with observations, based on transcriptome profiling, that βPG genes are differentially regulated during development [32] and stress treatments, for instance in *Glycine max* [33].

Didehydroamino acids have a double bond between the α and β carbon and are rarely found in proteins. One of the few known examples is the didehydroalanine found in the active site of phenylalanine ammonia lyase [34]. In proteome studies they are regularly reported as artefacts for instance after beta-elimination of phosphate from phosphoserine [35,36]. However, the here reported phenylalanine modification has a reproducibility and specificity as can be expected from enzymatic conversions. This substantiates that there is a previously-unknown enzymatic activity in plant cells converting Phe into ΔPhe, an activity similar to that of the LanB protein found in lantibiotic-synthesizing bacteria [37].

Dehydroamino acids are however identified in, and considered as an essential component of some bio-active peptides [38,39]. In these they force a stable, rigid conformation on small peptides such as lantibiotics and tentoxins. The introduction of dehydroamino acids in custom-made, often therapeutic, peptides was furthermore proposed to stabilize their conformation and make them resistant to proteolytic degradation [40]. Therefore conformational effects of dehydroamino acids, especially ΔPhe, in peptides were extensively studied [41]. In a recent review of results of such studies, the ability of ΔPhe to induce specific conformations (β-bends in small and 310-helices in larger peptides) is emphasized [42]. The impact ΔPhe has on the conformation of larger proteins was furthermore recently illustrated with the structural elucidation of a recombinant insulin beta chain [43] (http://www.rcsb.org/pdb/explore.do?structureId=2MLI).

*De novo* modelling of the protein fold was attempted in order to estimate the impact of the found modification on the fold of the protein. When the sequence of the active βPG without modifications is modelled using I-TASSER [44], a highly unstructured fold is predicted and this with a low confidence. A prediction that classifies the protein as an intrinsically unstructured protein [45,46]. No tool that allows the modelling of the protein fold with ΔPhe was found and trials with short sequence stretches were inconclusive. Furthermore a reliable estimation of the structural conformation induced by ΔPhe requires that the isomeric form of the modified amino acid, E or Z form, is known [47].

However, based on the conformation-determining effect of ΔPhe, it can be expected that the numerous ΔPhe in βPG force the polypeptide in a conformation that optimizes the interaction with its two known interaction partners. On one side the protein binds pectin, but it is also known to interact with the catalytic polygalacturonase subunit (PG2) forming the heterodimer PG1 [11]. While little is known about the interaction leading to the formation of PG1, protein-pectin interaction is known to occur either through positively charged residues or by the interaction of aromatic amino acid side chains with the polycarbohydrate [48,49]. Both of these interaction mechanisms could be used by βPG. The active form is mainly composed of 14 amino acid long repetitions starting with FxxY, it thus contains an unusual high percentage of aromatic amino acids: 14.2% compared to an 8.6% natural abundance of the aromatic amino acids. The position of the positively charged amino acids is furthermore relatively conserved among different species, and this especially around the FxxY repeats. Since no reliable structural prediction was obtained, either these interaction modes, or a combination of both, can contribute to the function of this protein.

The here described observations and the known conformational effects of dehydroamino acids potentially offer an alternative for the production of custom-folded proteins. The ΔPhe-containing peptides that are used to study the conformational effects of dehydrophenylalanine are produced by solid phase peptide synthesis, as is the insulin described by Menting et al. [43]. While improvements have been done to this approach it is still unsuited for any, other than laboratory-scale, application. Although requiring further study, the ability to use a cell-based system for the production of proteins with a custom-made, stabilized fold would offer great advantages.

## Experimental procedures

### Extraction of cell wall proteins from alfalfa stems

The cell wall proteins were extracted according to [18]. Briefly, 7 g of *M*. *sativa* (cv Giulia, SA Pinault Bio, Pleugueneuc, France) stems were ground in liquid nitrogen and a cell wall enriched fraction obtained using an increasing sucrose gradient (5 mM Na acetate pH 4.6, 4°C supplemented respectively with 0.4 M sucrose, 0.6 M sucrose or 1 M sucrose). The final cell wall pellet was washed twice with 5 mM Na acetate, pH 4.6. After washing, a sequential extraction of cell wall proteins was done with 5 mM Na acetate buffers at pH4.6 with 200 mM CaCl2, 50 mM EGTA and 3 M LiCl respectively. The CaCl_2_, EGTA and LiCl fractions were concentrated separately to a volume of approximately 200 μl (Amicon Ultra-15 10 K, Millipore) and further washed and desalted using the ReadyPrep 2-D Cleanup kit (Bio-Rad). Samples were solubilized in labelling buffer (7 M urea, 2 M thiourea, 2% CHAPS, 30 mM Tris) and protein concentrations were determined with the Bradford method.

Two-dimensional electrophoresis was done with DiGE labelling and the proteins separated on 3–10 NL strips as previously described [18]. After the second dimension and fixation (15% ethanol v/v, 1% m/v citric acid) overnight the gels were scanned (Typhoon FLA 9500 GE Healthcare), the images analysed and spots that changed significantly selected for identification (SameSpot software, TotalLab).

### Extraction of proteins from *Cannabis sativa* hypocotyls

Cannabis hypocotyls (cv. Santhica 27, CCPSC, Le Mans, France) were crushed to a fine powder in liquid nitrogen. Approximately 300 mg of material was homogenized in ice-cold extraction buffer (TCA 20%, DTT 0.1% in acetone) and proteins allowed to precipitate overnight at -20°C. After centrifugation (30000 g; 45 min; 4°C), the pellet was washed three times in ice-cold acetone, each time followed by a centrifugation step identical to the one described above, and vacuum dried. Proteins samples were solubilised in 500 μl labelling buffer (7 M urea, 2 M thiourea, 4% CHAPS, 30 mM Tris) for 30 min. After centrifugation (15000 g; 15 min; 4°C), the supernatants were transferred to a 1.5 mL tube and pH adjusted to 8.5 with sodium hydroxide. Protein concentration was determined using the 2-D Quant Kit (GE Healthcare) with BSA as standard. Following quantification, 50 μg protein was labelled with Cydyes (GEHealthcare) and 2D gels ran as described above.

### Identification of gel-separated proteins

Differentially abundant spots were picked and digested using the standard laboratory workflow [18]. Extracted peptides were dried, resolubilized in 2 μL of 50% v/v ACN containing 0.1% v/v TFA and 0.7 μL was spotted on a MALDI target. To this 0.7 μL α-cyano-4-hydroxycinnamic acid solution (7 mg/mL in 50% ACN/0.1% TFA (v/v)) was added and the samples were allowed to dry under ambient conditions. For each of the spots a MS spectrum was acquired and internally calibrated using trypsin autocleavage products. The ten highest precursors, excluding known contaminants, are automatically selected and fragmented, each MS/MS spectrum being the accumulation of 3000 shots. The MS spectrum and the MS/MS spectra were submitted together using an in-house MASCOT server (Matrix Science, www.matrixscience.com, London, U.K.). The databases used for alfalfa samples were alfalfa nucleotide sequences (http://plantgrn.noble.org/AGED/) containing 675756 sequences [32]. For identification of the *Cannabis sativa* proteins an in-house generated transcriptome was used (containing 170598 sequences). The following search parameters were used: mass tolerance MS 75 ppm, mass tolerance MS/MS 0.5Da, cysteine carbamidomethylation as fixed modification, and as variable modifications methionine oxidation, double oxidation of tryptophan, tryptophan to kynurenine and didehydrophenylalanine. Proteins were considered as identified when at least two peptides passed the MASCOT-calculated 0.05 threshold score of 40. All identifications reported here were manually validated.

### LC-MS/MS analysis of in-house generated cell wall extracts

Proteins in solution were digested with trypsin using Amicon Ultra-4 10K Centrifugal Filter Devices (Millipore) as previously performed [18,50]. Digested peptides were solubilized (45 μL 5% v/v ACN and 0.05% v/v TFA) and a sample of 5μl was desalted and concentrated on a C18 pre-column (C18 PepMapTM, 5 μm, 5 mm * 300 μm i.d., Thermo scientific, Bremen, Germany) prior to separation on a C18 reverse phase column (PepMapTM 100, 3 μm, 100Å, 75 μm id x 15 cm, Thermo scientific) using an Eksigent NanoLC-2D (Sciex, Darmstadt, Germany). Separation was performed at flow rate of 300 nl/min using a linear binary gradient (solvent A: 0.1% formic acid (FA); solvent B: 80% ACN 0.1% FA). Peptides were eluted for 50 min from 5% of solvent B to 55% of solvent B, afterwards the column was washed for 5 min with 100% of solvent B and re-equilibrated with 5% solvent B for 18 min.

Fragmentation spectra were acquired online with a Triple TOF 5600+ mass spectrometer (Sciex, Darmstadt, Germany) connected via a NanoSpray III source and a PicoTip® silica emitter of 10 μm i.d. (New Objective, Woburn, MA). Parameters of CID fragmentations for MS/MS spectra acquisitions were automatically adjusted by the system. The top 20 precursors ions of each MS scan were selected for MS/MS high sensitivity scan acquisition. The dynamic exclusion time for MS/MS acquisition was set at 10 s. The system was controlled by Analyst software (version TF1.7). Automatic mass recalibration was performed using digested beta-galactosidase as standard (LC-MS Peptide Calibration Kit, Sciex).

Proteins were identified with the in-house MASCOT server using the above mentioned databases. Settings were adapted for the identification of proteins after LC-MS/MS analysis.

### Reanalysis of downloaded datasets

Added to the in-house generated datasets from alfalfa and cannabis the datasets corresponding to published studies were reanalysed (Table 2). Some datasets were selected based on the degree of characterization of the genome/proteome of the species and on the known expression of βPG in the studied tissue.

The data-files in different formats were converted to mgf-files and these submitted in database searches using parameters mimicking those used in the original experiment, with the addition of ΔPhe as variable modification. Species-specific databases were downloaded from large depositories such as NCBI or from dedicated websites. After verification that the databases contain βPG homologs, these databases were used for protein identification as described above.

In the initial database searches with dataset PRD000044 completely different tools were used. Therefore significantly different parameters, compared to the original analysis, were used. This dataset was re-analysed with the following parameters: database NCBInr limited to *Arabidopsis*, average precursor mass, mass tolerance MS of 3Da, mass tolerance MS/MS 0.6Da, carbamidomethyl cysteine as fixed modification and oxidation of methionine and ΔPhe as variable modification.

Not all datasets linked to the articles on maize were reanalysed [22,23]. For each of the following tissues (endosperm, juvenile leaf, leaf, EZ, MZ, cortex) one complete dataset was randomly selected and analysed. Most of the database search parameters are identical to those used in the original studies by Walley *et al*., changes are the inclusion of ΔPhe as variable modification and the definition of the precursor m/z as average masses.

## Supporting information

S1 File **Figure A**. Data indicating that the identification of an unmodified phenylalanine in the peptide VNFVNYGQSFNPGSETFTGYGK is erroneous. **Table A**. All the peptides from the beta-subunit of polygalacturonase identified in this study.
(PDF)
