# Supplementary material for: Didehydrophenylalanine, an abundant modification in the beta subunit of plant polygalacturonases
Source: PLoS One. 2017 Feb 16;12(2):e0171990. doi: 10.1371/journal.pone.0171990 (PMC5313189; doi:10.1371/journal.pone.0171990)
Supplement: S1 File — Figure A. Data indicating that the identification of an unmodified phenylalanine in the peptide VNFVNYGQSFNPGSETFTGYGK is erroneous. Table A. All the peptides from the beta-subunit of polygalacturonase identified in this study. (PDF) [file pone.0171990.s001.pdf]

S1 File: **Figure A.** Data indicating that the identification of an unmodified phenylalanine in the peptide VNFVNYGQSFNPGSETFTGYGK is erroneous.

Dataset PRD000044 file 3337: calculated and matched fragment ions of the peptide VNFVNYGQSFNPGSETFTGYGK as identified by MASCOT using the selected doubly charged precursor of 1206.69. This resulted in the identification of the peptide with only the phenylalanine at position 17 identified as being modified. The fragments that were matched are indicated in bold red.

#	b	b^{++}	b*	b^0	b^{0++}	Seq.	y	y^{++}	y^{*++}	y^0	y^{0++}	#
1	100.0757	50.5415				V						22
2	214.1186	107.5629	197.0921			N	2312.026	1156.516	1148.003	2294.015	1147.511	21
3	**361.187**	181.0972	344.1605			F	2197.983	1099.495	1090.982	2179.972	1090.49	20
4	**460.2554**	230.6314	443.2289			V	2050.914	1025.961	1017.447	2032.904	1016.955	19
5	574.2984	287.6528	557.2718			N	***1951.846***	976.4265	967.9132	1933.835	967.4212	18
6	**737.3617**	369.1845	720.3352			Y	***1837.803***	919.405	910.8917	1819.792	910.3997	17
7	794.3832	397.6952	777.3566			G	***1674.739***	837.8734	829.3601	1656.729	828.8681	16
8	**922.4417**	461.7245	905.4152			Q	***1617.718***	809.3626	800.8494	1599.707	800.3573	15
9	**1009.474**	505.2405	992.4472	991.4632	496.2352	S	***1489.659***	745.3333	736.8201	1471.649	736.3281	14
10	1156.542	578.7747	1139.516	1138.532	569.7694	F	***1402.627***	701.8173	693.3041	1384.617	692.812	13
11	**1270.585**	635.7962	1253.559	1252.575	626.7909	N	***1255.559***	628.2831	619.7698	1237.548	619.2778	12
12	1367.638	684.3226	1350.611	1349.627	675.3173	P	***1141.516***	571.2617	562.7484	1123.506	562.2564	11
13	1424.659	712.8333	1407.633	1406.649	703.828	G	***1044.463***	522.7353	514.222	1026.453	513.73	10
14	1511.691	756.3493	1494.665	1493.681	747.344	S	***987.4418***	494.2245	485.7113	969.4312	485.2193	9
15	**1640.734**	820.8706	1623.707	1622.723	811.8653	E	***900.4098***	450.7085	442.1953	882.3992	441.7032	8
16	**1741.782**	871.3945	1724.755	1723.771	862.3892	T	***771.3672***	386.1872	377.674	753.3566	377.1819	7
17	1886.834	943.9208	1869.808	1868.824	934.9156	F	***670.3195***	335.6634	327.1501	652.3089	326.6581	6
18	1987.882	994.4447	1970.856	1969.872	985.4394	T	***525.2667***	263.137	254.6237	507.2562	254.1317	5
19	2044.904	1022.955	2027.877	2026.893	1013.95	G	***424.2191***	212.6132	204.0999			4
20	2207.967	1104.487	2190.94	2189.956	1095.482	Y	***367.1976***	184.1024	175.5892			3
21	2264.988	1132.998	2247.962	2246.978	1123.993	G	204.1343	102.5708	94.0575			2
22						K	147.1128	74.06	65.5468			1

The presence of an unmodified phenylalanine at position 3 is however not in agreement with the details of the MS/MS spectrum.

In the table above the b(8) ion is matched at m/z 922.4, however a peak at m/z 920.5 would be a better match.

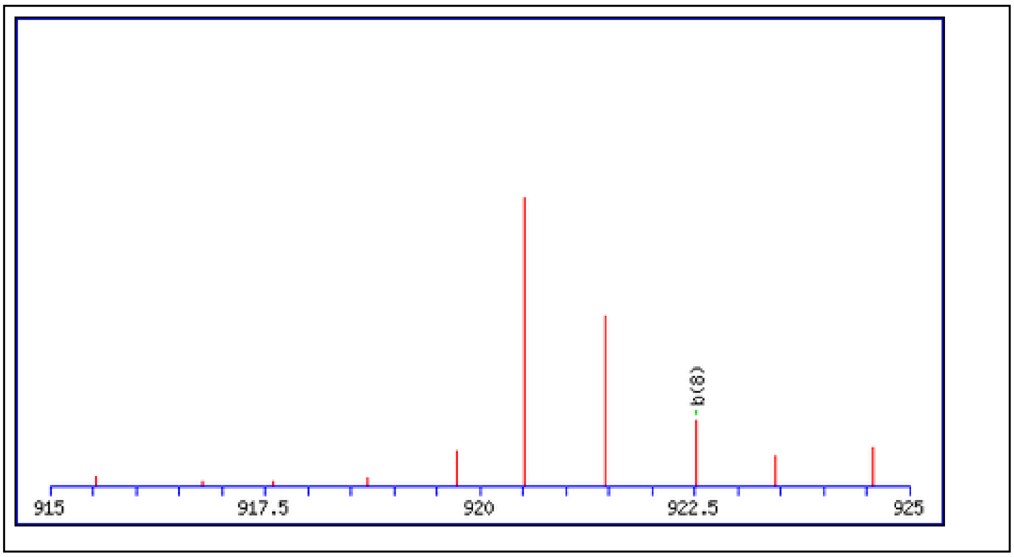

For the b(9) ion matched at 1009.5, a more intense peak is actually observed at 1007.5

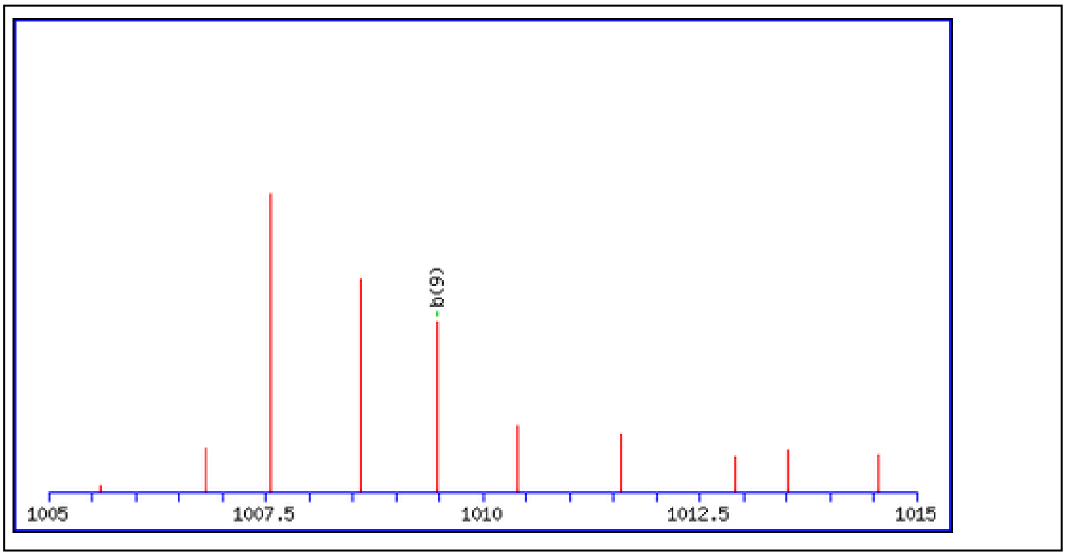

For the b(11) ion matched at 1270.6 a more intense peak is observed at 1268.5

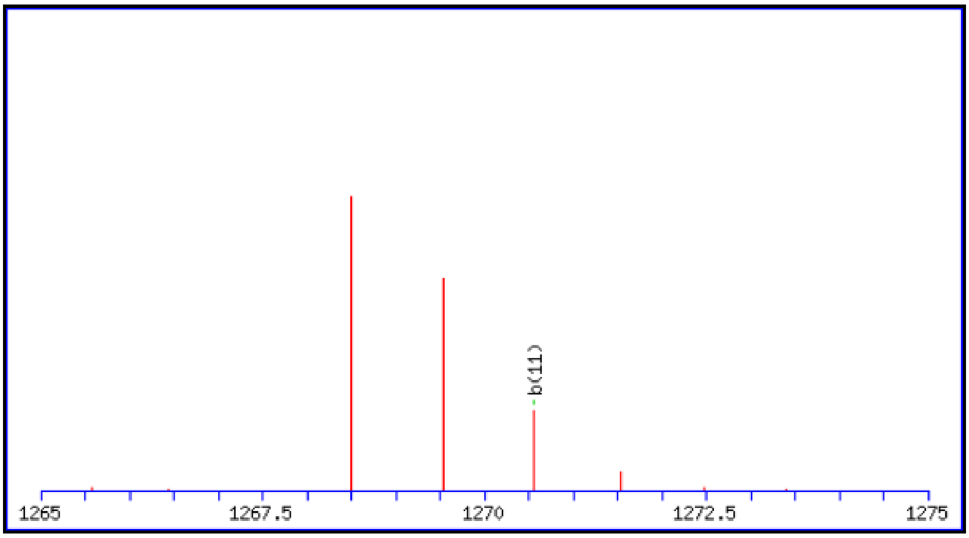

These three details of the MS/MS spectrum and the observation that the isotopic cluster of these fragments do not show any deviations from what can be expected indicate that contrary to the match-table the phenylalanine at position three (VN**F**VNYGQSFNPGSETFTGYGK) is indeed modified and shows a mass loss of two Dalton.

Using the y-ion series, the same can be concluded for the phenylalanine at position 17

The y(5) ion does not contain a phenylalanine and its mass matched exactly the one found in the above match-table.

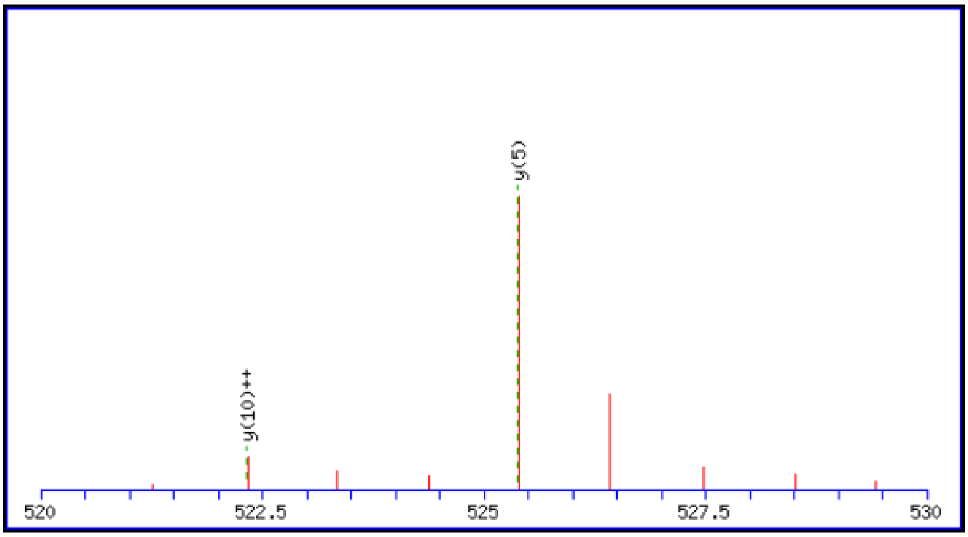

The y(6) ion contains a phenylalanine, modified based on the database search, and indeed the m/z of the observed ion is in agreement with the predicted y(6) for a modified phenylalanine.

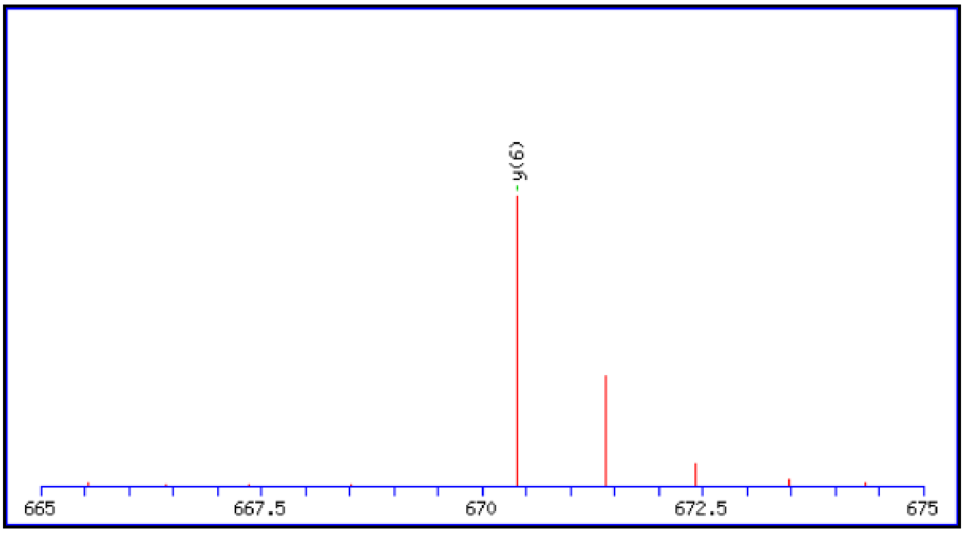

The y(7) ion is again in agreement with the predicted m/z.

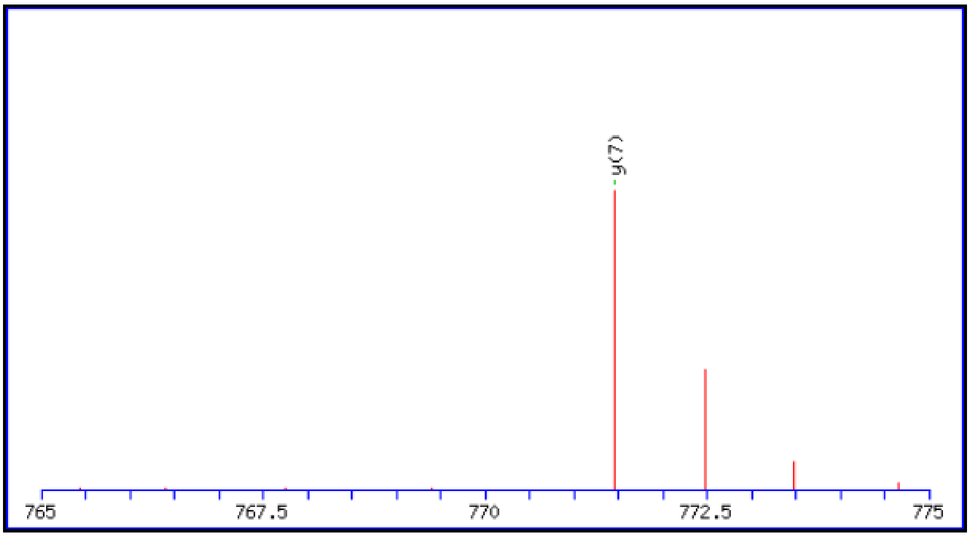

For larger y-ions this match stays good, as for instance for the y(16) ion.

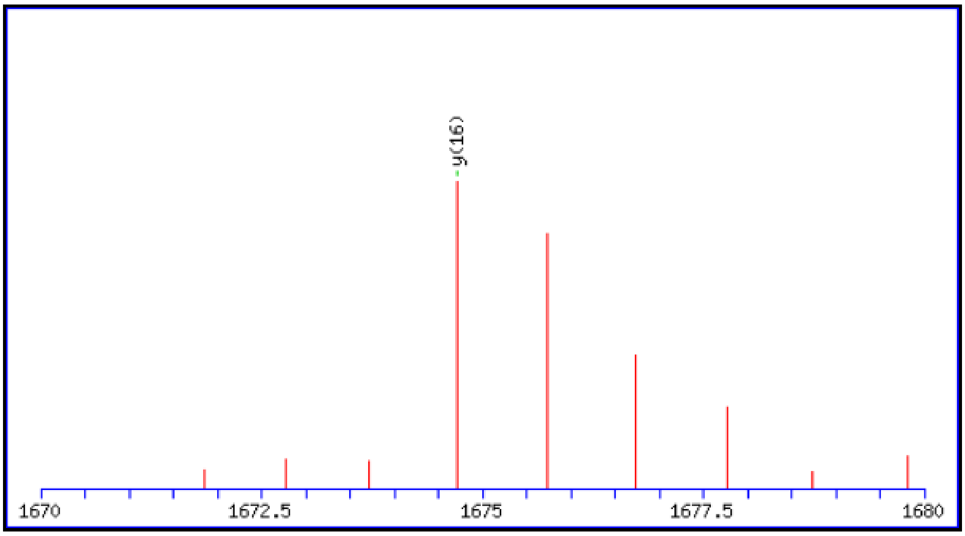

The same is the case for the entire y-ion series. Since there is furthermore no significant shift in the isotopic distribution (in casus an unexpected increase of the peak at the monoisotopic mass plus 2), based on these spectra it must be concluded that the phenylalanine at position 17 (VNFVNYGQSFNPGSET**F**TGYGK) is quantitatively converted into didehydrophenylalanine.

The peak list of the corresponding MS/MS spectrum was isolated from the result files and searched individually. The precursor mass used in the database search was calculated based on the summation of corresponding y- and b-ions. For the dataset 3337 this results in a corrected precursor m/z of 1205.69 when doubly charged. The use of this corrected precursor mass resulted in an

increase of the score of the search from 63 to 77 (as given in table 1 in the manuscript) for the peptide with both phenylalanines modified, the match table for the latter result is given below.

#	b	b^{++}	b*	b^{0}	b^{0++}	Seq.	y	y^{++}	y^{*++}	y^{0}	y^{0++}	#
1	100.0757	50.5415				V						22
2	214.1186	107.5629	197.0921			N	2310.01	1155.509	1146.995	2291.999	1146.503	21
3	359.1714	180.0893	342.1448			F	2195.967	1098.487	1089.974	2177.956	1089.482	20
4	458.2398	229.6235	441.2132			V	2050.914	1025.961	1017.447	2032.904	1016.955	19
5	572.2827	286.645	555.2562			N	***1951.846***	976.4265	967.9132	1933.835	967.4212	18
6	735.3461	368.1767	718.3195			Y	***1837.803***	919.405	910.8917	1819.792	910.3997	17
7	792.3675	396.6874	775.341			G	1674.739	837.8734	829.3601	1656.729	828.8681	16
8	**920.4261**	460.7167	903.3995			Q	1617.718	809.3626	800.8494	1599.707	800.3573	15
9	1007.458	504.2327	990.4316	989.4476	495.2274	S	***1489.659***	745.3333	736.8201	1471.649	736.3281	14
10	1154.527	577.7669	1137.5	1136.516	568.7616	F	***1402.627***	701.8173	693.3041	1384.617	692.812	13
11	**1268.57**	634.7884	1251.543	1250.559	625.7831	N	***1255.559***	628.2831	619.7698	1237.548	619.2778	12
12	1365.622	683.3148	1348.596	1347.612	674.3095	P	***1141.516***	571.2617	562.7484	1123.506	562.2564	11
13	1422.644	711.8255	1405.617	1404.633	702.8202	G	1044.463	522.7353	514.222	1026.453	513.73	10
14	1509.676	755.3415	1492.649	1491.665	746.3362	S	***987.4418***	494.2245	485.7113	969.4312	485.2193	9
15	**1638.718**	819.8628	1621.692	1620.708	810.8575	E	***900.4098***	450.7085	442.1953	882.3992	441.7032	8
16	**1739.766**	870.3866	1722.739	1721.755	861.3814	T	***771.3672***	386.1872	377.674	753.3566	377.1819	7
17	**1884.819**	942.913	1867.792	1866.808	933.9077	F	***670.3195***	335.6634	327.1501	652.3089	326.6581	6
18	**1985.866**	993.4369	1968.84	1967.856	984.4316	T	***525.2667***	263.137	254.6237	507.2562	254.1317	5
19	2042.888	1021.948	2025.861	2024.877	1012.942	G	***424.2191***	212.6132	204.0999			4
20	2205.951	1103.479	2188.925	2187.941	1094.474	Y	***367.1976***	184.1024	175.5892			3
21	2262.973	1131.99	2245.946	2244.962	1122.985	G	204.1343	102.5708	94.0575			2
22						K	147.1128	74.06	65.5468			1

The most probable reason is a combination of different things. First of all the data was acquired on a mass spectrometer with a relatively low resolution. For higher m/z precursors this may result in the loss of isotopic resolution and thus the selection of a precursor that in mass approaches the average peptide mass and not the monoisotopic. While this in general will pose little problem, in our case the mass shift induced by the conversion of phenylalanine to didehydrophenylalanine is lower than the mass error allowed for the search.

S1 File: Table A. all the peptides from the beta-subunit of polygalacturonase identified in this study.

The data from the 4 species wherein the protein and its dehydrated phenylalanine was found are grouped.
Note that when a peptide was found using both MALDI and ESI from internal or external datasets, it is only added once.
The annotation of the alfalfa contigs is linked to the Alfalfa gene index and expression atlas database (http://plantgrn.noble.org/AGED/)
Note: The contig 53836 contains two repetitions of beta-polygalacturonase, one homologous to contig 12933 and a second with lower homology

Alfalfa (in-house generated data and Verdonk et al. Front Plant Sci (2012) 3, art.: 279)

contig_12933 — the same set of peptides also found in contig_111493, contig_22692, contig_27771, contig_48302, contig_51002, contig_53836

Obs	Exp	Cal	Error (ppm)	Score	peptide sequence
346.69	691.37	691.37	3.29	29	K.NANVFK.S
378.19	754.36	754.36	0.72	33	K.GADLDHK.V
509.21	1016.41	1016.41	-0.27	36	K.SDFSGYSEK.G + Didehydro (F)
1149.55	1148.54	1148.54	4.74	42	K.HNEFARYSK.I + Didehydro (F)
1285.60	1284.60	1284.60	-2.26	87	K.SPGSETFKGYAK.G + Didehydro (F); Oxidation (P)
1315.61	1314.60	1314.61	-3.41	84	K.GSSAVDAFVSYSK.N + Didehydro (F)
1548.74	1547.73	1547.74	-2.42	97	K.DSIHAVNGFTQYAK.N + Didehydro (F)
1653.75	1652.74	1652.75	-2.22	75	K.VDFNNYGLNPDYPK.S + Didehydro (F)
1664.84	1663.83	1663.83	-1.3	102	K.NLNKPENTFKNYGK.G + Didehydro (F)
2314.01	2313.01	2313.01	-0.4	151	K.SDFSGYSEKGSSAVDAFVSYSK.N + 2 Didehydro (F)
2920.35	2919.34	2919.34	1.91	45	K.VDFNNYGLNPDYPKSPGSETFKGYAK.G + 2 Didehydro (F); Oxidation (P)
2987.38	2986.37	2986.36	2.89	109	K.NANVFKSDFSGYSEKGSSAVDAFVSYSK.N + 2 Didehydro (F)
1728.83	1727.83	1727.83	0	77	L.NKPENTFKNYGKGSF.A + Didehydro (F)

the same set of peptides also found in contig_111493, contig_22692, contig_27771, contig_48302, contig_51002

Obs	Exp	Cal	Error (ppm)	Score	peptide sequence
2161.92	2160.91	2160.91	1.08	182	R.DGAGSGDDSFTSYGESSQLGVK.V + Didehydro (F)

the same set of peptides also found in contig_22692, contig_27771, contig_48302

Obs	Exp	Cal	Error (ppm)	Score	peptide sequence
1516.72	1515.71	1515.71	0.39	90	K.GSFAAQEKFTNYR.D + Didehydro (F)
2075.91	2074.90	2074.90	0.8	27	K.GSFAAQEKFTNYRDGAGSGD + Didehydro (F)
3659.69	3658.68	3658.61	19.5	93	K.GSFAAQEKFTNYRDGAGSGDDSFTSYGESSQLGVK.V + 2 Didehydro (F)
3084.34	3083.33	3083.30	10.4	67	F.AAQEKFTNYRDGAGSGDDSFTSYGESSQL.G + 2 Didehydro (F)

contig_51001

Obs	Exp	Cal	Error (ppm)	Score	peptide sequence
779.35	1556.68	1556.67	0.69	63	R.DQANVGADSFSSYAK.D + Didehydro (F)
805.84	1609.67	1609.66	2.74	79	K.DSTGGTHVDFDNYGK.S + Didehydro (F)

Obs	Exp	Cal	Error (ppm)	Score	peptide sequence
818.35	2452.02	2452.02	-0.52	53	R.QQTFTSYSEAGNAGDQSFGNYGK.D + 2 Didehydro (F)
988.80	2963.36	2963.37	-2.06	46	R.GSLGAVEKFSNYRDQANVGADSFSSYAK.D + 2 Didehydro (F)

contig_53836

Obs	Exp	Cal	Error (ppm)	Score	peptide sequence
471.24	940.47	940.47	-0.18	33	K.GISFAGYTK.K + Didehydro (F)
535.29	1068.56	1068.56	-2.66	36	K.KGISFAGYTK.K + Didehydro (F)
713.36	1424.71	1424.70	1.1	38	R.GSLGAVEKFSNYR.D + Didehydro (F)
516.91	1547.69	1547.69	3.12	38	K.SFNEGTDSFKGYAK.G + Didehydro (F)
779.34	1556.67	1556.67	-0.42	79	R.DQANVGADSFSSYAK.D + Didehydro (F)
805.84	1609.67	1609.66	2.74	79	K.DSTGGTHVDFDNYGK.S + Didehydro (F)
689.02	2064.04	2064.04	2.43	53	K.DTNVPNLRFTTYSVGVAGR.Q + Didehydro (F)
694.35	2080.03	2080.03	-1.06	56	K.DTNVPNLRFTTYSVGVAGR.Q + Didehydro (F); Oxidation (P)
818.35	2452.02	2452.02	-0.12	44	R.QQTFTSYSEAGNAGDQSFGNYGK.D + 2 Didehydro (F)
988.80	2963.37	2963.37	-0.36	41	R.GSLGAVEKFSNYRDQANVGADSFSSYAK.D + 2 Didehydro (F)

the same set of peptides also found in contig_111493

Obs	Exp	Cal	Error (ppm)	Score	peptide sequence
1532.71	1531.70	1531.71	-0.54	72	K.GSFASQEKFTNYR.D + Didehydro (F)

contig_93293

the same set of peptides also found in contig_101380

Obs	Exp	Cal	Error (ppm)	Score	peptide sequence
456.22	910.42	910.42	0.18	37	K.IGFDNYGK.S + Didehydro (F)
318.18	951.52	951.52	1.88	45	K.VGFKGYGVK.N + Didehydro (F)
514.74	1027.46	1027.46	-2.93	28	K.NDFKEYSK.E + Didehydro (F)
672.81	1343.60	1343.60	-1.71	87	K.SNVGADSFTSYAK.T + Didehydro (F)
783.35	1564.68	1564.67	5.44	54	K.SFNEGTDTFTSYAK.T + Didehydro (F)
843.36	2527.06	2527.05	3.76	39	R.TQSFNSYTENGNSGQQSFTSYGK.N + 2 Didehydro (F)

contig_65436

Obs	Exp	Cal	Error (ppm)	Score	peptide sequence
434.24	866.47	866.47	1.32	30	R.AFKVYNK.N + Didehydro (F)
615.28	1228.54	1228.54	0.05	44	K.SDSNFAVYSNK.K + Didehydro (F)
728.82	1455.63	1455.63	-0.18	59	R.ANVGDDSFQSYGAK.S + Didehydro (F)
1100.00	2197.99	2197.99	-2.17	103	K.SSFKAYGTGSVSGSDTFVSYR.N + 2 Didehydro (F)

contig_94188

	Obs	Exp	Cal	Error (ppm)	Score	peptide sequence
	822.68	2465.02	2465.02	0.45	30	R.QQTFNSYSEAGNAGDQSFGNYGK.D + 2 Didehydro (F)

Cannabis sativa

6D1_S2_L001_R1_001_(paired)_contig_648 Mass: 91038 Score: 96 Expect: 0.00037 Matches: 1 Frame: 2

ired)_contig_648 polygalacturonase isoenzyme 1 beta subunit homolog

	Obs	Exp	Cal	Error (ppm)	Score	peptide sequence
	1548.69	1547.69	1547.69	-2.67	101	K.SFNEGTDKFTGYGK.G + Didehydro (F)
	1583.71	1582.70	1582.70	-0.28	77	K.SSNAEQINFNNYGK.S + Didehydro (F)
	3000.38	2999.38	2999.39	-5.18	29	R.QGGSDQFKNYSPGENIPVDSFRRYSR.D + 2 Didehydro (F)

***Arabidopdis thaliana* (Pride: PRD000044; Baerenfaller et al. Science (2008) 320, 938-941)**

gi|1762584 polygalacturonase isoenzyme 1 beta subunit homolog [Arabidopsis thaliana]

	Obs	Exp	Cal	Error (Da)	Score	peptide sequence
dataset 3322	1205.80	2409.59	2408.07	1.5145	70	K.VNFVNYGQSFNPGSETFTGYGK.G + 2 Didehydro (F)
dataset 3335	1205.01	2408.01	2408.07	-0.0655	46	K.VNFVNYGQSFNPGSETFTGYGK.G + 2 Didehydro (F)
dataset 3337	1205.69	2409.36	2408.07	1.2943	77	K.VNFVNYGQSFNPGSETFTGYGK.G + 2 Didehydro (F)

***Arabidopdis thaliana* (De Vijlder et al. J Chromatography (2015) 974, 48-56)**

gi|1762584 polygalacturonase isoenzyme 1 beta subunit homolog [Arabidopsis thaliana]

	Obs	Exp	Cal	Error (ppm)	Score	peptide sequence
Fraction 10	679.80	1357.58	1357.58	4.46	49	K.ANVGDDSFSSYAK.D + Didehydro (F)

***Zea mays* (Walley et al. PNAS (2013) 110, E4808-4817 & Walley et al. Science (2016) 353, 814-818)**

Dataset: Maize_endosperm-10-try-150ug-2D29-122109-LTQ3 (22;23)

gi|195613864 polygalacturonase-1 non-catalytic beta subunit precursor [Zea mays]

Fraction	Obs	Exp	Cal	Error (Da)	Score	peptide sequence
20	553.59	1657.75	1657.65	0.098	40	R.DDGNVGDDRFTSYAK.G + ΔPhe
	829.75	1657.49	1657.65	-0.16	48	R.DDGNVGDDRFTSYAK.G + ΔPhe
23, 24 & 25	1117.84	2233.67	2233.22	0.44	108	R.SFASYSQEANHGENGFSGYGK.N + 2 ΔPhe
	745.83	2234.47	2233.22	1.25	60	R.SFASYSQEANHGENGFSGYGK.N + 2 ΔPhe
27	326.11	650.21	649.31	0.89	30	K.SGVDFK.G + ΔPhe
28	693.23	2076.67	2076.19	0.48	46	FRSYGAGGNAGVDTFKNYR + 2 ΔPhe

Dataset Maize juvenileleaf-1-Try-3mg-MCX-1500ugCeO2-Elu-2d19-030210-LTQ3

gi|195613864 polygalacturonase-1 non-catalytic beta subunit precursor [Zea mays]

Fraction	**Obs**	**Exp**	**Cal**	**Error (Da)**	**Score**	**peptide sequence**
14	554.13	1659.28	1657.65	1.63	28	R.DDGNVGDDRFTSYAK.G + ΔPhe
	830.12	1658.23	1657.65	0.58	43	R.DDGNVGDDRFTSYAK.G + ΔPhe
17 & 18	1117.86	2233.71	2233.22	0.48	80	R.SFASYSQEANHGENGFSGYGK.N + 2 ΔPhe
